# Influenza hospitalisations in Spain between the last influenza and COVID-19 pandemic (2009–2019)

**DOI:** 10.1017/S0950268823001620

**Published:** 2023-10-04

**Authors:** Javier Canelas-Fernández, Clara Mazagatos, Concepción Delgado-Sanz, Amparo Larrauri

**Affiliations:** 1 Universidad de Cantabria Facultad de Medicina, Medicina Preventiva, Santander, Spain; 2 National Centre of Epidemiology, CIBERESP, Carlos III Health Institute, Madrid, Spain

**Keywords:** Influenza, Public health, Surveillance, Burden of disease

## Abstract

Knowing the burden of severe disease caused by influenza is essential for disease risk communication, to understand the true impact of vaccination programmes and to guide public health and disease control measures. We estimated the number of influenza-attributable hospitalisations in Spain during the 2010–2011 to 2019–2020 seasons – based on the hospitalisations due to severe acute respiratory infection (SARI) in Spain using the hospital discharge database and virological influenza information from the Spanish Influenza Sentinel Surveillance System (SISSS). The weekly numbers of influenza-attributable hospitalisations were calculated by multiplying the weekly SARI hospitalisations by the weekly influenza virus positivity, obtained from the SISSS in each season, stratified by age group and sex. The influenza-related hospitalisation burden is age-specific and varies significantly by influenza season. People aged 65 and over yielded the highest average influenza-attributable hospitalisation rates per season (615.6 per 100,000), followed by children aged under 5 (251.2 per 100,000). These results provide an essential contribution to influenza control and to improving existing vaccination programmes, as well as to the optimisation and planning of health resources and policies.

## Introduction

Seasonal influenza epidemics present a considerable disease burden, causing a high number of hospitalisations and deaths, as well as significant resource consumption worldwide [1]. The latest estimates from the World Health Organization (WHO) indicate that there are between 3 and 5 million severe cases annually, with an estimated number of influenza-related deaths ranging from 290,000 to 650,000 worldwide, depending on the season [2]. In Europe, influenza is associated with 5 million infections with mild symptoms, 150,000 hospitalisations, and 15,000 to 40,000 deaths annually [3]. These estimates are similar to those of the GLaMOR Project, which found that 27,600 respiratory deaths were associated with seasonal influenza in 28 EU countries per winter [4].

The most effective methods to prevent and control the influenza virus, within those considered acceptable to implement, are influenza vaccination programmes [5]. The WHO publishes twice-yearly recommendations on the strains to include in the influenza vaccine for the upcoming season, one in February for the Northern Hemisphere [6] and another in September for the Southern Hemisphere [7]. These recommendations aim to facilitate the creation of vaccines with viruses as similar as possible to those circulating in the population recently. The European Centre for Disease Prevention and Control (ECDC) also publishes national influenza vaccination recommendations and vaccination coverage rates for the 27 member states [8]. However, despite national and international recommendations, influenza vaccination coverage remains low and vaccination coverage for 65 years and over in 22 European countries was well below the 75% threshold recommended by the Council of the European Union for the 2007–2008 to 2017–2018 seasons [8, 9].

The influenza vaccine was introduced in Spain in the early 1980s [10]. Currently, the Interterritorial Council of the National Health System (CISNS) recommends influenza vaccination for those aged 65 and over and those aged 6 months or older at high risk of complications from influenza [11]. Even so, Spain’s flu vaccination coverage has decreased in recent years from 57.7% in the 2011–2012 season to 54.7% in 2019–2020 [12].

One of the reasons for this low coverage is a lack of knowledge about the actual severity of influenza and its disease burden [13]. Knowing the burden of severe disease caused by influenza is essential for disease risk communication, understanding the true impact of vaccination programmes, and guiding public health and disease control measures [13–15].

In America, the Centers for Disease Control and Prevention (CDC) publishes annual estimates of the burden of disease caused by influenza in terms of mild illness, hospitalisations, and influenza-associated deaths in the United States. In the 2021–2022 season, the CDC estimated 9,000,000 influenza illnesses, 4,000,000 medical visits for flu, 100,000 flu-attributable hospitalisations, and 5,000 deaths [16].

Other examples of studies that estimate hospitalisations and deaths attributable to influenza have been conducted throughout the Americas [13] and in the United States [17], Argentina [18], Finland [19], Russia [20], or Portugal [21].

In Spain, the National Center of Epidemiology (CNE) at the Carlos III Health Institute estimates the seasonal burden of influenza based on the information provided by the influenza surveillance systems, including the number of cases of mild influenza attended to in primary care, hospitalisations, and admissions to intensive care units (ICUs) with confirmed influenza and all-cause excess mortality attributable to influenza. During the 2017–2018 to 2019–2020 seasons, influenza epidemics were produced between 28,000 and 52,000 hospitalisations with influenza [22], updating previously published estimates [23]. However, disease burden estimates from surveillance systems have limitations and may underestimate Spain’s true influenza burden. Hospital influenza surveillance systems are based on reporting hospitalised cases that have been laboratory-confirmed as influenza. As the number of confirmed influenza hospitalisations reported depends directly on the swabbing policy in each participating hospital, this can lead to an underestimation of the real number of influenza hospitalisations, making the estimation of hospitalisations attributable to seasonal influenza epidemics a real challenge [13, 16, 23].

Some authors have approached this question using different methodologies, not based on hospital surveillance systems but on information from hospital discharge databases in combination with influenza circulation data from surveillance systems [13, 14, 24].

In Spain, we have a very reliable influenza sentinel surveillance system (Sistema Centinela de Vigilancia de Gripe en España (SISSS)), which in the 2019–2020 season included 555 primary care physicians and 217 paediatricians and monitored 2.4% of the population of the seventeen autonomous communities (CCAA) that use the influenza sentinel surveillance networks [25]. Furthermore, since 1996 and until the emergence of SARS-CoV-2 in 2020, the SISSS has provided timely epidemiological and virological information on the pandemic [26] and seasonal influenza activity in Spain [25], with consistent epidemiological and virological surveillance indicators at national and regional levels.

Based on the estimated hospitalisations due to severe acute respiratory infection (SARI) in Spain using the hospital discharge database and the influenza virological information from the SISSS, this study aimed to estimate the number of influenza-attributable hospitalisations in Spain across the 2010–2011 to 2019–2020 seasons.

## Methods

### Population and study period

Our study included all patients hospitalised for a severe acute respiratory infection (SARI) during the 2010–2011 to 2019–2020 influenza seasons in Spain.

### Study design and information sources

We conducted a retrospective observational ecological study using discharge records from the Minimum Basic Data Set (CMBD) and information from the SISSS.

The CMBD is a hospital discharge database covering approximately 98% of public hospitals in Spain, although the number of private hospitals included has increased since 2005, with 85% of the discharge diagnostic data corresponding to public hospitals in 2018 [27]. Diagnoses were coded using the International Classification of Diseases (ICD) 9th revision, clinical modification (ICD-9) until 1 January 2016 and the ICD-10-CM since.

The weekly percentage of influenza virus positivity was obtained from the SISSS as the ratio between the number of laboratory-confirmed influenza cases and the respiratory swabs from the influenza-like illness (ILI) cases visiting sentinel physicians in primary care. Weekly influenza positivity was calculated for all ages and for the 0–4, 5–14, 15–64, and 65 and over age groups in each of the ten influenza seasons studied: 2010–2011 to 2019–2020.

### Definition of SARI hospitalisation

A SARI hospitalisation was defined as an episode with any of the ICD codes listed in Table 1 included in the principal diagnosis. These ICD codes indicative of SARI were defined in the ‘Protocol for hospital-based, test-negative case–control studies to measure seasonal influenza vaccine effectiveness against laboratory-confirmed influenza SARI hospitalization’ [28], recently updated by the ECDC [29].

**Table 1.** SARI-related ICD codes^a^

Diagnosis category	Sign or symptom	ICD-9	ICD-10
Influenza-like illness	Cough	786.2	R05
	Difficulty breathing	786.05	R06
	Sore throat	784.1	R07.0
	Dysphagia	787.20	R13
	Fever	780.6	R50.9
	Headache	784	R51
	Myalgia	729.1	M79.1
	Fatigue/malaise	780.79	R53.1 R53.81 R53.83
Cardiovascular	Acute myocardial infarction or acute coronary syndrome	410–411, 413–414	I20–23 I24–25
	Heart failure	428–429.0	I50, I51
Respiratory	Emphysema	492	J43.9
	Chronic obstructive pulmonary disease	496	J44.9
	Asthma	493	J45
	Myalgia	786.0	R06.0
	Dyspnoea/respiratory abnormality	786.00	R06.9
	Respiratory abnormality	786.05	R06.02
	Shortness of breath	786.09	R06.00 R06.09 R06.3 R06.89
Infections	Pneumonia and influenza	480–488.1	J09-J18
	Other acute lower respiratory infections	466, 519.8	J20-J22
	Viral infection, unspecified	790.8	B34.9
	Bacterial infection, unspecified	041.9	A49.9
	Bronchitis	490, 491	J40, 41
Inflammation	SIRS noninfectious without acute organ dysfunction	995,94	R65.11
	SIRS noninfectious with acute organ dysfunction	995,93	R65.10
Deterioration of general condition or functional status	General physical deterioration, lethargy, tiredness	780,79	R53.1 R53.81 R53.83
	Anorexia	783.0	R63.0
	Feeding difficulties	783,3	R63.3
	Abnormal weight loss	783.21	R63.4
	Other symptoms and signs concerning food and fluid intake	783.9	R63.8
	Disorientation/altered mental status	780.97	R41.0
	Dizziness and giddiness	780.4	R42
	Infective delirium	293.0, 293.1	F05
	Coma	780.01	R40.2
	Transient alteration of awareness	780.02	R40.4
	Other alterations of consciousness (somnolence, stupor)	780.09	R40.0 R40.1
	Febrile convulsions (simple), unspecified	780.31	R56.00
	Complex febrile convulsions	780.32	R56.01

Abbreviation: ICD, International Statistical Classification of Diseases and Related Health Problems.

a Based on ref. [28, 29].

### Definition of the influenza season

An influenza season is when the flu virus is expected to circulate with greater intensity than in the rest of the year. In Spain, as a temperate country in the Northern Hemisphere, this period begins in epidemiological week 40 of one year and ends in week 20 of the following, from approximately October to May.

### Data analysis

**Figure 1.**
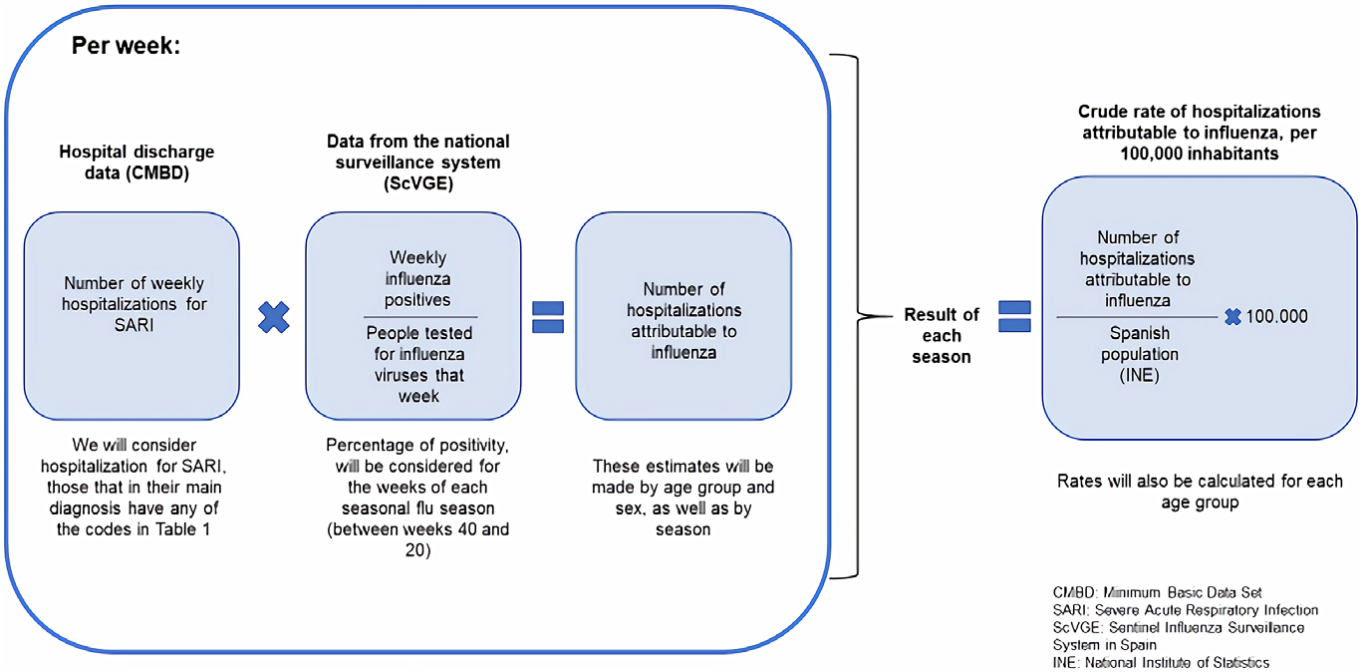
Scheme of the method followed to estimate the number and rate of hospitalisations attributable to influenza.

During the influenza seasons studied, the weekly number of SARI hospitalisations was estimated for all ages and for the 0–4, 5–14, 15–64, and 65 and over age groups. To calculate the weekly number of influenza-attributable hospitalisations, we multiplied the weekly SARI hospitalisations by the weekly influenza virus positivity percentage in each season, obtained from the SISSS (Figure 1) by age group and sex. The total number of influenza-attributable hospitalisations by age group in each season was calculated with the sum of the respective weekly estimates. We divided this number by the corresponding population in the analysed group to obtain the hospitalisation rates attributable to influenza by sex and for all ages and the analysed age groups in each influenza season (Figure 1). Crude rates are shown as SARI-attributable hospitalisations per 100,000 population. We used the exact method to calculate the 95% confidence intervals (CIs), assuming that influenza-related hospitalisations follow a Poisson distribution.

Population data by age and sex were obtained from the Spanish Statistical Office (INE). For each season, we used the population corresponding to the second year of the influenza season as the denominator.

We used the statistical package Stata 16.1 and Microsoft Excel 2013 for data treatment.

This study did not require the approval of an ethics committee, as the data obtained from hospital discharge and influenza surveillance systems are anonymised. Therefore, they did not include any personal identification codes or other personal information.

## Results

### Number of SARI hospitalisations by season and age group

The highest number of SARI hospital admissions occurred in the 2019–2020 season (303,781 hospitalisations), followed by 2017–2018 (277,831) and 2016–2017 (267,297) (Table 2). By age group, the highest number of hospitalisations occurred in the 65 and over age group, with the highest number of SARI hospitalisations in the 2017–2018 season (198,670 hospitalisations) (Table 2). In the 15 and under age group, the differences in the number of SARI hospitalisations between seasons were smaller (Table 2). For the 0–4 age group, seasonal SARI hospitalisations increased from 2015 to 2016 onwards (Table 2).

**Table 2.** Number of hospitalisations for severe acute respiratory infections (SARIs) by season and age group. Spain, seasons 2010–2011 to 2019–2020

Season	0–4 years	5–14 years	15–64 years	≥65 years	Total
2010–2011	13,690	4,030	47,423	161,185	226,328
2011–2012	10,965	2,879	41,477	171,516	226,837
2012–2013	9,957	3,206	43,809	165,118	222,090
2013–2014	10,064	3,269	44,459	168,053	225,845
2014–2015	10,431	3,864	46,318	197,176	257,789
2015–2016	16,770	2,992	48,402	179,695	247,859
2016–2017	22,396	2,122	45,975	196,804	267,297
2017–2018	24,467	2,371	52,323	198,670	277,831
2018–2019	24,635	2,704	50,858	172,633	250,830
2019–2020	21,518	2,412	88,968	190,883	303,781

### Percentage of sentinel samples positive for influenza viruses by season and age group

During the study period, A(H1N1)pdm09 was the dominant strain in three influenza seasons (2010–2011, 2015–2016, and 2019–2020), A(H3N2) in another three (2011–2012, 2014–2015, and 2016–2017), influenza B in 2012–2013, and a mix of circulating viruses was observed in 2013–2014, 2017–2018, and 2018–2019 (Table 3).

**Table 3.** Percentage of sentinel samples positive for influenza viruses by season and age group. Spain, seasons 2010–2011 to 2019–2020

Season	Dominant influenza virus	0–4 years	5–14 years	15–64 years	≥65 years	All ages
2010–2011	A(H1N1)pdm09	37.6%	50.1%	45.9%	25.7%	45.0%
2011–2012	A(H3N2)	47.9%	51.8%	49.2%	56.8%	50.0%
2012–2013	B	39.8%	58.8%	52.5%	36.2%	51.4%
2013–2014	A(H1N1)pdm09/(A(H3N2)	45.1%	47.7%	53.4%	43.0%	50.3%
2014–2015	A(H3N2)	40.9%	61.9%	53.8%	54.6%	54.5%
2015–2016	A(H1N1)pdm09	46. 8%	55.8%	51.7%	39.6%	51.2%
2016–2017	A(H3N2)	36.3%	51.4%	49.4%	45.6%	47.8%
2017–2018	B/A(H3N2)	52.0%	62.2%	57.6%	59.0%	58.1%
2018–2019	A(H3N2)/ A(H1N1)pdm09	46.7%	59.0%	50.1%	46.4%	51.5%
2019–2020	A(H1N1)pdm09	49.1%	67.0%	50.4%	34.5%	53.7%
Season average		44.2%	56.6%	51.4%	44.1%	51.4%

Sentinel respiratory influenza sample positivity for each season studied and by age group is shown in Table 3. The average seasonal positivity for all ages was 51.4%, ranging from 45.0% in the 2010–2011 season to 58.2% in 2017–2018 (Table 3). By age group, the 5–14 age group yielded the highest positivity, with 67.0% in the 2019–2020 season and 62.2% in 2017–2018. 65 and over (59.0%) and under 5 years (52.0%) had the highest positivities in the 2017–2018 season (Table 3).

### Weekly SARI hospitalisations and influenza-attributable hospitalisations by age group

**Figure 2.**
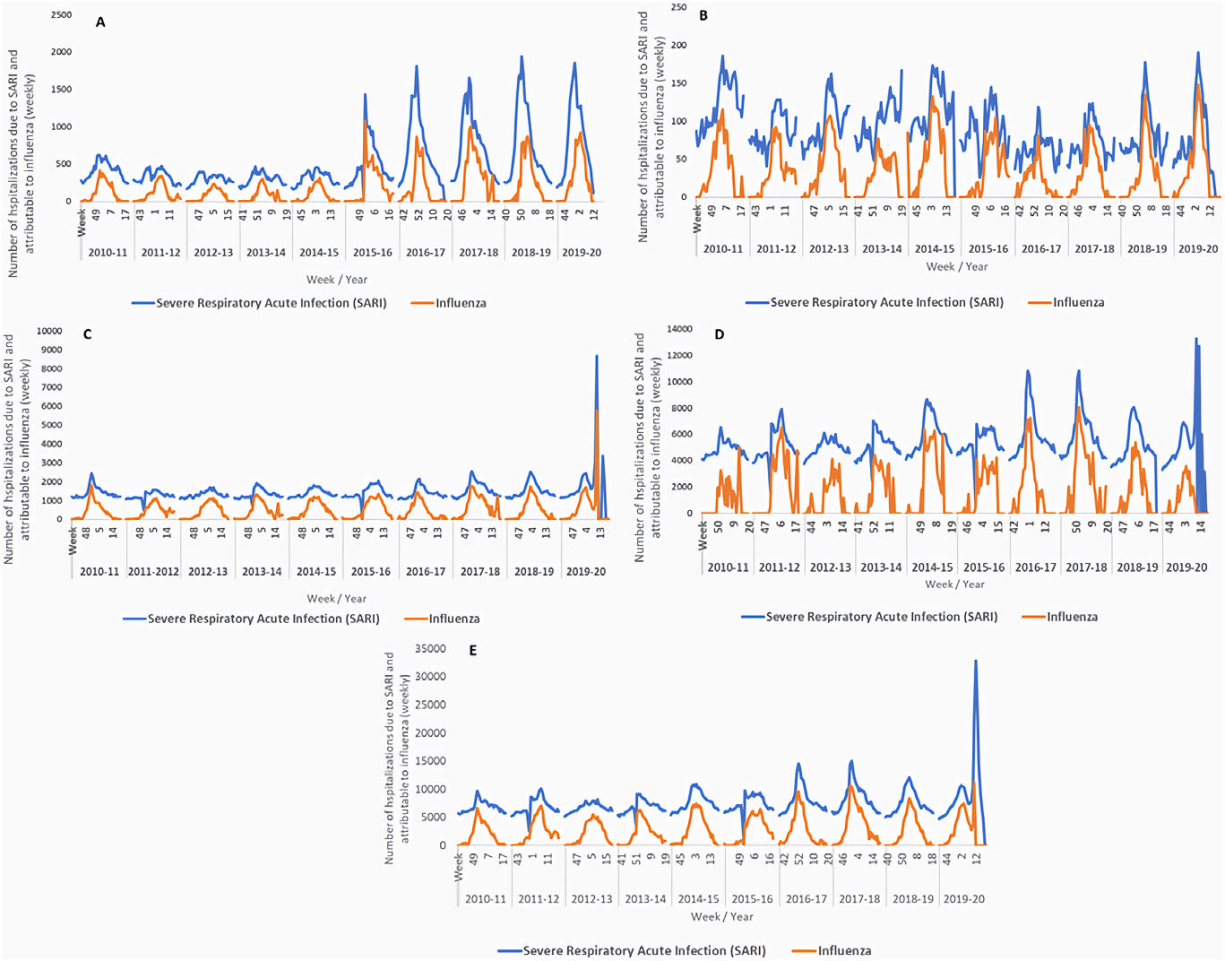
Weekly number of SARI hospitalisations and hospitalisations attributable to influenza by age group. Spain, seasons 2010–2011 to 2019–2020. A) 0-4 years, B) 5-14 years, C) 15-64 years, D) ≥65 years and E) All ages.

The weekly distribution of SARI hospitalisations and influenza-attributable hospitalisations, during the ten influenza seasons studied, by age group and for all ages, is shown in Figure 2. Both followed the same pattern over time, with hospitalisations peaking between the last weeks of one year and the first weeks of the following year.

In the 0–4 age group, the highest weekly number of SARI hospitalisations was observed in the 2018–2019 season (1,943 SARI hospitalisations in week 51/2018), followed by 2019–2020 (1,862 SARI hospitalisations in week 51/2019), while influenza-attributable hospitalisations reached a high in the 2015–2016 season (1,076 hospitalisations in week 53/2015), followed by the 2017–2018 season (1,006 hospitalisations in week 52/2017) (Figure 2a). In the first five seasons of the study (2010–2011 to 2014–2015), the estimated number of influenza-attributable SARI hospitalisations was considerably lower than in the rest of the studied seasons (Figure 2a).

In the 5–14 age group, we estimated a lower number of weekly SARI hospitalisations during the study period, not reaching 200 SARI hospitalisations in any week, nor more than 150 attributable to influenza (Figure 2b).

In the 15–64 age group, SARI hospitalisations were estimated at 1,000–1,500 hospitalisations per week. The 2019–2020 season was the one that featured the highest number of hospitalisations attributable to influenza, reaching 5,787 in week 11/2020 (Figure 2c).

The 65 and over age group yielded the highest weekly hospitalisation numbers, for both SARI and influenza (Figure 2d). In this age group, the number of SARI hospitalisations reached almost 4,000 in every week of the seasons studied (Figure 2d). The highest estimated weekly numbers of influenza-attributable hospitalisations were found in the 2011–2012, 2014–2015, 2016–2017, and 2017–2018 seasons (Figure 2d).

The weekly number of SARI hospitalisations for all ages presented a maximum that ranged between 6,000 and 8,000 hospitalisations (Figure 2e), with the most affected seasons similar to those noted for the 65 and over age group (Figure 2e).

### Weekly influenza-attributable hospitalisations by sex

**Figure 3.**
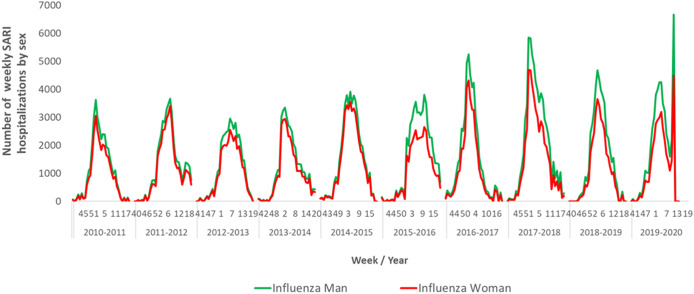
Number of weekly hospitalisations attributable to influenza by sex. Spain, seasons 2010–2011 to 2019–2020.

The number of weekly hospitalisations attributable to influenza was higher in men than in women in all seasons studied, with higher differences in the periods of maximum influenza activity (Figure 3).

### Number and seasonal average influenza-attributable hospitalisation rates by season and age group

A seasonal average of 84,551 influenza-attributable hospitalisations was estimated, ranging from 65,089 in the 2010–2011 season to 118,716 hospitalisations in 2017–2018 (Table 4). Those seasons had minimum and maximum influenza-attributable hospitalisation rates of 141.5 and 256.4 hospitalisations per 100,000 inhabitants.

**Table 4.** Number and crude rates of hospitalisations attributable to influenza by season and age group. Spain, 2010–2011 to 2019–2020 season

	0–4 years		5–14 years		15–64 years		≥65 years		All ages	
Season	N	Rate (95% CI)	N	Rate (95% CI)	N	Rate (95% CI)	N	Rate (95% CI)	N	Rate (95% CI)
2010–2011	3,934	161.3 (156.3–166.4)	1,248	27.9 (26.4–29.5)	14,278	45.7 (45.0–46.5)	31,603	402.8 (398.4–407.3)	65,089	141.5 (140.4–142.6)
2011–2012	3,467	138.8 (134.2–143.5)	1,064	23.6 (22.2–25.0)	14,099	44.5 (43.8–45.3)	68,925	863.4 (857.0–869.9)	80,356	172.2 (171.0–173.4)
2012–2013	2,453	101.0 (97.0–105.0)	1,286	28.1 (26.6–29.6)	14,291	45.5 (44.7–46.2)	37,315	459.5 (454.9–464.2)	71,474	153.5 (152.4–154.7)
2013–2014	2,530	107.1 (102.9–111.3)	965	20.8 (19.5–22.1)	15,339	49.2 (48.4–50.0)	46,960	568.0 (562.9–573.2)	71,505	153.9 (152.7–155.0)
2014–2015	2,815	122.5 (118.0–127.1)	1,540	32.6 (31.0–34.3)	16,455	53.1 (52.3–53.9)	67,726	804.0 (798.0–810.1)	93,530	201.4 (200.1–202.7)
2015–2016	7,165	320.4 (313.0–327.9)	1,178	24.6 (23.2–26.0)	17,353	56.3 (55.5–57.2)	50,338	586.1 (580.1–591.2)	90,053	193.9 (192.7–195.2)
2016–2017	5,805	269.8 (262.9–276.9)	686	14.2 (13.2–15.3)	14,526	47.3 (46.6–48.1)	59,382	681.6 (676.2–687.2)	87,179	188.0 (186.7–189.3)
2017–2018	9,852	477.6 (468.2–487.1)	1,003	20.7 (19.5–22.1)	22,848	74.8 (73.8–75.7)	81,099	917.4 (911.1–923.8)	118,716	256.4 (254.9–257.9)
2018–2019	7,783	392.8 (384.1–401.6)	1,023	21.2 (19.9–22.5)	16,385	53.8 (53.0–54.7)	48,956	544.2 (39.4–549.0)	82,464	178.4 (177.1–179.6)
2019–2020	8,078	421.0 (411.9–430.3)	1,261	26.4 (25.0–27.9)	20,563	67.8 (66.9–68.7)	30,134	329.4 (325.7–333.1)	85,143	184.4 (183.2–185.7)
Season average	5,338	251.2 (244.9–257.7)	1,125	24.0 (22.6–25.4)	16,614	53.8 (53.0–54.6)	52,244	615.6 (610.4–620.9)	84,551	182.4 (181.29–183.6)

By age group, the seasonal average number of influenza-attributable hospitalisations was estimated at 52,244 for 65 years and over, 16,614 for 15- to-64-year-olds, 1,125 for 5- to 14-year-olds, and 5,388 for under 5 years (Table 4).

**Figure 4.**
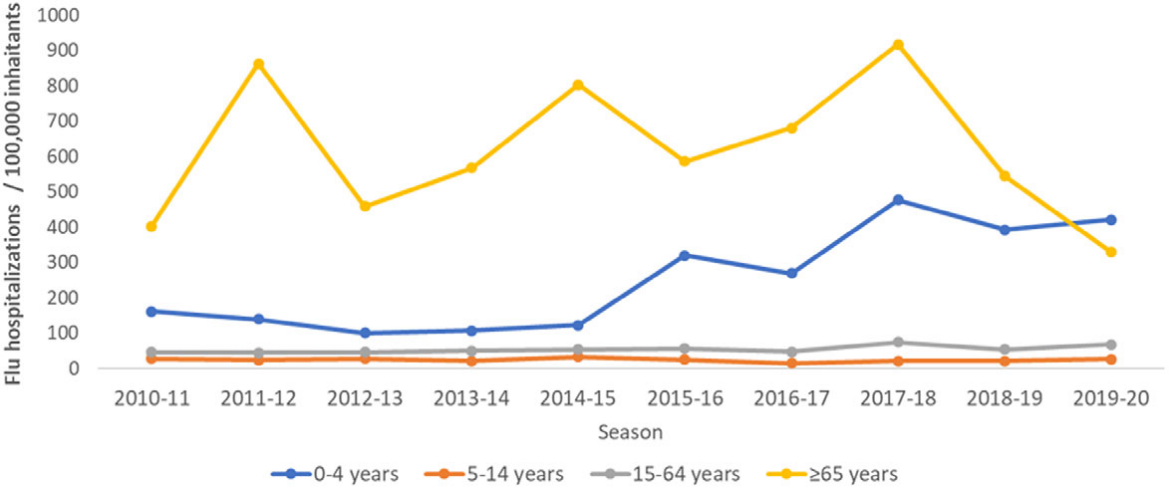
Crude rates of hospitalisations attributable to influenza by season and age group. Spain, seasons 2010–2011 to 2019–2020.

The highest hospitalisation rates were estimated for 65 years and over in all seasons studied, with maximum rates for this age group found in the 2011–2012, 2014–2015, and 2017–2018 influenza seasons (Figure 4 and Table 4). Average seasonal hospitalisation rates attributable to influenza were estimated at 615.6 per 100,000 inhabitants for 65 years and over, 251.2 for those under 5 years, 24.0 for 5- to 14-year-olds, and, finally, 53.8 per 100,000 inhabitants for 15- to 64-year-olds (Table 4 and Figure 4).

Higher differences in hospitalisations attributable to influenza between seasons were observed in the 0–4 and 65 and over age groups. For the 0–4 age group, rates ranged from 101.0 hospitalisations per 100,000 in 2012–2013 to 477.6 in 2017–2018. In the 65 and over age group, rates ranged from 402.8 in 2010–2011 to 917.4 in 2017–2018 (Table 3 and Figure 4).

## Discussion

We estimated the burden of influenza-attributable hospitalisations in Spain during the 2010–2011 to 2019–2020 seasons. An estimated seasonal average of nearly 85,000 influenza-attributable hospitalisations was found, with an average annual rate of 182.4 influenza-attributable hospitalisations per 100,000 population.

In this study, we used data from the CMBD and SISSS to identify the respiratory pathologies of patients discharged from hospital and the influenza virus positivity during each influenza season in Spain. Similar approaches have been used to estimate the impact of influenza on hospitals in Portugal, estimating 5,107–6,742 hospitalisations for 65 years and over during the 2014–2015 to 2016–2017 seasons [14]. Another study conducted in 16 countries across the Americas between 2010 and 2015 reported a range of 287 to 734 hospitalisations per 100,000 inhabitants in the United States alone, for the 65 and over age group from 2010 to 2013 [13]. These figures are similar to our estimates of 402.8 to 917.4 hospitalisations per 100,000 for this age group. However, other countries showed greater differences than our results, such as Cuba, with 7–46 hospitalisations attributable to influenza per 100,000 inhabitants [13]. These differences are likely due to different countries’ hospitalisation policies, codes used to select SARI, and types of surveillance systems. Previous studies in Spain estimated a range of 147 to 247 influenza-attributable hospitalisations per 100,000 inhabitants for the 2000 to 2004 seasons [24], which is in a very similar range to the results of the present study (141.5 to 256.4 influenza-attributable hospitalisations per 100,000 population), despite the use of different SARI codes and seasons.

Our results showed that the burden of influenza-related hospitalisations is age-specific and varies significantly by influenza season. Persons aged 65 and over presented the highest hospitalisation rates (917.4 hospitalisations per 100,000), consistent with having the highest risk of developing complications from influenza [3, 30]. The second highest number of estimated hospitalisations attributable to influenza, although far lower than that for the elderly, was for children aged under 5 (477.6 hospitalisations per 100,000). A similar pattern of influenza-attributable hospitalisations by age has been reported in previous studies conducted in the Americas [13], with 141 for 65 years and over and 90 for children aged under 5 influenza-attributable hospitalisation rates; the United States with 309.1 and 151.0 [17]; Finland with 47.3 and 15.2 [19]; and Chile with 156.0 and 71.5 [31]. Different studies in Spain also showed the highest number of influenza-attributable hospitalisations in those aged 65 and over and in children aged under 5 [23, 24, 32]. Differences found in the influenza-attributable hospitalisation rates as compared to our study might be due to differences in surveillance systems, the use of different codes to select SARI, or even differences between countries in the criteria for hospitalisation, reflecting the specific health systems in place in each country.

In contrast, another study conducted in Spain estimated the highest admission rates (60.1 per 100,000 inhabitants) for children aged under 5 between 2009 and 2015 [33]. In a study conducted in England, almost 40% of influenza-attributable hospitalisations were in children aged under 15 [34].

In our study, we estimated the highest all-age rate of influenza-attributable hopitalisations in the season 2017–2018. In this season, the influenza activity was mainly associated with the circulation of a B Yamagata virus, not included in the influenza vaccine for that year, which correlated with unexpected all-cause excess mortality [25, 35]. In the 2014–2015 season, we observed the second highest all-age rate of influenza-attributable hospitalisations, at 201.4 influenza-attributable hospitalisations per 100,000 population. The predominant influenza virus circulating in Spain that season was A(H3N2) with a B virus circulating at the end of the epidemic period [36]. In addition, this season recorded the highest cumulative influenza rate since the 2009 pandemic, suggesting high disease transmissibility [25].

The influenza hospitalisation burden is different for each age group in each season. In the 65 and over age group, the highest hospital burden occurred in 2017–2018 with 917.4 influenza-attributable hospitalisations per 100,000, followed by 2011–2012 with 863.4 and 2014–2015 with 804.0. Surveillance of severe hospitalised confirmed influenza cases also attributed the highest hospitalisation rates to the 65 and over age group in those seasons [25]. These results coincide with the dominance of the A(H3N2) virus, which has been associated with a heavy impact on both hospitalisations and deaths attributable to influenza in the elderly [37, 38].

In the 5–14 age group, the highest estimated influenza-related hospitalisation rates were in seasons with significant influenza B circulation. This virus was dominant in Spain in the 2012–2013 season and presented late activity peaks in the 2014–2015 and 2015–2016 seasons [25]. In 2015–2016, virtually all confirmed influenza hospitalisations reported to the severe influenza surveillance system in that age group were due to influenza B. Several studies have confirmed that influenza B predominantly affects the 5–14 age group. A meta-analysis conducted in twenty-nine countries in different world regions between 1999 and 2014 found that infection with B influenza was the most frequent among older children [37]. Another study concluded that influenza B generally affected younger people than influenza A, especially school-aged children (5 to 17) and adults (18 to 64) [39]. Similar results were also found in other studies conducted in Germany and Italy [40, 41].

Children aged under 5 had the highest hospitalisation rates in the 2017–2018 and 2019–2020 seasons, when influenza B was either dominant (B Yamagata in 2017–2018) or co-circulating with other influenza viruses, such as A(H1N1)pdm09 in 2018–2019 [25]. These results suggest the potential for influenza B to cause serious illness among young children and are consistent with previous data from hospital surveillance in Spain that show high hospitalisation rates for 5 years in the 2017–2018 (34.7/100,000) and 2019–2020 seasons (52.4/100,000) [25]. However, differences in influenza-related hospitalisation across seasons in under 5 years should be interpreted with caution. The change in clinical discharge diagnostic codes from ICD-9 to ICD-10 in January 2016 could have led to higher numbers of specific and unspecific SARI infections in children being reported. The difference might also be attributable to the lower number of SARI hospitalisations in the under 5 years before the 2015–2016 season. In the 15–64 age group, although slightly higher in 2017–2018 than in other years, no notable differences were found in hospitalisation rates across the different seasons.

Hospitalisation distribution by sex presents a higher influenza hospitalisation burden in men than women throughout the study period, with the greatest differences observed in peak activity weeks. However, we found mixed evidence for how sex may influence influenza disease burden estimates. For example, some studies consider being a woman a protective factor for the most critically ill patients [42]; others, depending on the type of influenza virus involved, claim that more cases are reported in adult woman age groups, probably linked to differences in health-seeking behaviour [43].

This study has several limitations. First, as with previous studies using similar estimation approaches, we have conducted an ecological study that utilises different data sources, which limits the study’s external validity [14]. In addition, using different case definitions, estimation methods, and data sources limits the possible comparison between our results and those obtained in other influenza hospitalisation burden studies. In fact, differences in the SARI codes used for the extraction of clinical episodes potentially related to influenza are extensive. Some authors included ICD respiratory discharge-coded hospitalisations in principal diagnostics [13]; other SARI codes correspond to influenza, viral or unspecified pneumonia, bacterial pneumonia, febrile convulsions, and acute respiratory distress syndrome (ARDS) [19]. In a previous Spanish study, they even used ICD codes for pneumonia (due to all viral, bacterial, and unknown causes), chronic bronchitis, heart failure, and influenza present in all possible diagnostics [24]. Remarkably, the use of respiratory and non-respiratory SARI codes is very frequent in influenza hospitalisation burden studies. However, by including all SARI codes listed in Table 1, we might ourselves be overestimating the influenza burden in hospitals. However, we only recorded the diagnostics included in the CMBD as principal diagnoses, in contrast to other authors who also used secondary diagnoses. In addition, data from the CMBD hospital discharge records do not include laboratory information, only the coding of diagnoses and some procedures. This lack of information hinders the ability to estimate the actual number of hospitalisations attributable to influenza and leads to the necessity of using the external SISSS database to obtain an estimate of hospitalised influenza cases [14, 24]. Information about readmissions [24, 33] is not included either, which could overestimate the impact of influenza admissions. However, as these limitations are present in all seasons, our results should be reasonably comparable across the influenza seasons studied. In addition, although the delay between influenza community circulation and influenza hospitalisations is variable across the seasons and does not occur in all of them, it is possible that in some influenza seasons not applying a potential delay might somehow modify the estimated burden of hospitalisations attributable to influenza. Furthermore, the higher number of SARI hospitalisations in the 2019–2020 season, from March 2020, especially in individuals aged 15 years and over is likely related to the emergence of the COVID-19 pandemic. This increase might contribute to an overestimation of the influenza hospitalisation burden in the last part of the 2019–20 season.

Finally, the virological surveillance information from the SISSS only captures influenza cases that access the health system, usually with respiratory symptoms. However, it does not consider cases in the community that are asymptomatic or those with milder symptoms that do not access the health system. This is a general limitation of using influenza surveillance data sources for disease burden estimates.

The study also has several important strengths. First, the weekly integration of hospital discharge records with influenza surveillance data. These two different data sources are easily accessible, and therefore, the study could be replicated in subsequent seasons. Second, the method used to estimate the influenza-attributable hospitalisation burden is comparable to that of other authors who have estimated this burden in the Americas [13], Portugal [14], and Spain [24]. Third, the use of hospitalisations due to acute respiratory infections and correction for influenza virus circulation improves the specificity of the influenza hospitalisation estimates [14]. Finally, this analysis is an alternative to that previously used in Spain [23]. It is important to note that, based on hospital influenza surveillance systems in Spain, the National Centre for Epidemiology has estimated between 28,000 and 52,000 confirmed influenza hospitalisations per annum in persons 65 and over [22] during the 2017–2018 to 2019–2020 seasons, which is comparable to the estimated annual average of 52,000 influenza hospitalisations presented in this study. This comparison substantiates the results obtained by this study.

To conclude, our study provides evidence of the high influenza-attributable hospitalisation burden in Spain during the ten-season period between 2010–2011 and 2019–2020 by age, sex, and seasonal influenza epidemic. People aged 65 years and over and children aged under 5 have the highest hospitalisation rates, as observed in other countries and regions. These influenza burden estimates are continuously updated and are essential to understanding annual influenza epidemics’ real impact on populations and healthcare systems. Additionally, having burden estimates available by age group and season is critical when assessing the impact of influenza vaccination programmes and the benefits of increasing vaccination coverage in the population, especially in groups at high risk of influenza complications. Based on the high burden of influenza hospitalisation for the elderly in Spain, any public health measure that contributes to an increase in vaccine coverage for this age group and for other high-risk groups should be prioritised. The results of this study can provide an essential contribution to help guide public health measures for influenza control and for improving existing vaccination programmes, as well as aiding the optimisation and planning of health resources and policies.

## Data Availability

The data sets generated and analysed during this study are not publicly available due to restrictions imposed by the National Epidemiological Surveillance Network, but are available from the corresponding author upon reasonable request.

## References

[1] World Health Organization. Influenza (seasonal) factsheet. Available at http://www.who.int/en/news-room/fact-sheets/detail/influenza-(seasonal).

[2] Iuliano AD, et al.(2018) Estimates of global seasonal influenza-associated respiratory mortality: A modelling study. Lancet 391, 1285–1300. 29248255 10.1016/S0140-6736(17)33293-2 PMC5935243

[3] Rizzo C, Rezza G and Ricciardi W (2018) Strategies in recommending influenza vaccination in Europe and US. Human Vaccines & Immunotherapeutics 14, 693–698. 28922083 10.1080/21645515.2017.1367463 PMC5861797

[4] Paget J, et al. (2022) Estimates of mortality associated with seasonal influenza for the European Union from the GLaMOR project. Vaccine 40, 1361–1369. 35094868 10.1016/j.vaccine.2021.11.080 PMC8923032

[5] Paules C and Subbarao K (2017) Influenza. Lancet 390, 697–708. 28302313 10.1016/S0140-6736(17)30129-0

[6] World Health Organization. *Recommended composition of influenza virus vaccines for use in the 2023–2024 northern hemisphere influenza season.* Available at https://www.who.int/publications/m/item/recommended-composition-of-influenza-virus-vaccines-for-use-in-the-2023-2024-northern-hemisphere-influenza-season (accessed 9 May 2023).

[7] World Health Organization. *Recommended composition of influenza virus vaccines for use in the 2023 southern hemisphere influenza season.* Available at https://www.who.int/publications/m/item/recommended-composition-of-influenza-virus-vaccines-for-use-in-the-2023-southern-hemisphere-influenza-season (accessed 9 May 2023).

[8] European Centre for Disease Prevention and Control (2018) Seasonal influenza vaccination and antiviral use in EU/EEA Member States–Overview of vaccine recommendations for 2017–2018 and vaccination coverage rates for 2015–2016 and 2016–2017 influenza seasons. Published online: 2018.

[9] European Centre for Disease Prevention and Control*. Seasonal Influenza Vaccination in Europe: Vaccination Recommendations and Coverage Rates in the EU Member States for Eight Influenza Seasons 2007–2008 to 2014–2015. LU.* Publications Office: 2017.

[10] Mayo Montero E, et al. (2004) Evolución de las coberturas vacunales antigripales entre 1993–2001 en España: Análisis por Comunidades Autónomas. Revista Española de Salud Pública 78, 481–492. Published online: August 2004. 10.1590/S1135-57272004000400006. 15384262

[11] Ministerio de Sanidad and Gobierno de España (2022) *Recomendaciones de vacunación frente a la gripe. Temporada 2022–2023.*

[12] Ministerio de Sanidad. *Evolución de cobertura de vacunación frente a la gripe en población ≥ 65 años. España, temporadas de 2011–2012 a 2019–2020.*

[13] Palekar RS, et al. (2019) Burden of influenza-associated respiratory hospitalizations in the Americas, 2010–2015. PLoS One 14, e0221479. 31490961 10.1371/journal.pone.0221479 PMC6730873

[14] Machado A, et al. (2019) Impact of national influenza vaccination strategy in severe influenza outcomes among the high-risk Portuguese population. BMC Public Health 19, 1690. 31842831 10.1186/s12889-019-7958-8 PMC6916191

[15] Rolfes MA, et al. (2018) Annual estimates of the burden of seasonal influenza in the United States: A tool for strengthening influenza surveillance and preparedness. Influenza and Other Respiratory Viruses 12, 132–137. 29446233 10.1111/irv.12486 PMC5818346

[16] Centers for Disease Control and Prevention (CDC). *Disease Burden of Flu in the U.S. from 2010–2020.* Available at https://www.cdc.gov/flu/about/burden/index.html.

[17] Zhou H, et al. (2012) Hospitalizations associated with influenza and respiratory syncytial virus in the United States, 1993–2008. Clinical Infectious Diseases 54, 1427–1436. 22495079 10.1093/cid/cis211 PMC3334364

[18] Azziz-Baumgartner E, et al. (2013) Incidence of influenza-associated mortality and hospitalizations in Argentina during 2002–2009: Influenza mortality and hospitalizations. Influenza and Other Respiratory Viruses 7, 710–717. 23210456 10.1111/irv.12022 PMC5855154

[19] Jacks A, et al. (2012) Influenza-associated hospitalisations in Finland from 1996 to 2010: Unexpected age-specific burden during the influenza A(H1N1)pdm09 pandemic from 2009 to 2010. Euro Surveillance 17, 8. 23040966

[20] Goldstein E (2019) *Influenza-associated mortality for different causes of death during the 2010–2011 through the 2014–2015 influenza seasons in Russia.* Epidemiology.

[21] Rodrigues E, et al. (2018) Excess pneumonia and influenza hospitalizations associated with influenza epidemics in Portugal from season 1998/1999 to 2014/2015. Influenza and Other Respiratory Viruses 12, 153–160. 29460423 10.1111/irv.12501 PMC5818339

[22] Instituto de Salud Carlos III. *Carga de enfermedad de la gripe estacional e impacto de la vacuna antigripal. Infografías, temporadas 2017–2018 a 2019–2020.* Available at https://www.isciii.es/QueHacemos/Servicios/VigilanciaSaludPublicaRENAVE/EnfermedadesTransmisibles/Paginas/Infograf%C3%ADas.aspx.

[23] Oliva J, et al. (2018) Estimating the burden of seasonal influenza in Spain from surveillance of mild and severe influenza disease, 2010–2016. Influenza and Other Respiratory Viruses 12, 161–170. 28960828 10.1111/irv.12499 PMC5818358

[24] Lenglet AD, et al. (2007) Impact of flu on hospital admissions during 4 flu seasons in Spain, 2000–2004. BMC Public Health 7, 197. 17686175 10.1186/1471-2458-7-197 PMC1964764

[25] Instituto de Salud Carlos III. *Sistema de Vigilancia de Gripe en España. Informes anuales, temporadas 1999/2000–2019–2020 (Influenza Surveillance in Spain. Annual reports, seasons 1999/2000–2019–2020).* Available at https://www.isciii.es/QueHacemos/Servicios/VigilanciaSaludPublicaRENAVE/EnfermedadesTransmisibles/Paginas/Informes-anuales.aspx.

[26] Larrauri Cámara A, et al. (2012) Epidemiology of the 2009 influenza pandemic in Spain. The Spanish influenza surveillance system. Enfermedades Infecciosas y Microbiología Clínica 30, 2–9. 10.1016/S0213-005X(12)70098-8 23116786

[27] Ministerio de Sanidad. *Registro de Actividad Sanitaria Especializada (RAE-CMBD): Actividad y resultados de la hospitalización en el SNS. Año 2018.*

[28] I-MOVE+ (2019) *Protocol for hospital-based test negative case control studies to measure seasonal influenza vaccine effectiveness against influenza laboratory confirmed SARI hospitalisation among the elderly across the European Union and European Economic Area Member States.*

[29] European Centre for Disease Prevention and Control. *Core protocol for ECDC studies of COVID-19 vaccine effectiveness against hospitalisation with Severe Acute Respiratory Infection, laboratory-confirmed with SARS-CoV-2 or with seasonal influenza - Version 2.0.* Available at https://www.ecdc.europa.eu/en/publications-data/core-protocol-ecdc-studies-covid-19-vaccine-effectiveness-against-0 (accessed 28 May 2023).

[30] Smetana J, et al. (2018) Influenza vaccination in the elderly. Human Vaccines & Immunotherapeutics 14, 540–549. 28708957 10.1080/21645515.2017.1343226 PMC5861798

[31] Sotomayor V, et al. (2018) Estimating the burden of influenza-associated hospitalizations and deaths in Chile during 2012–2014. Influenza and Other Respiratory Viruses 12, 138–145. 29446231 10.1111/irv.12502 PMC5818356

[32] Pérez-Rubio A, Platero L and Eiros Bouza JM (2019) Gripe estacional en España: carga clínica y económica y programas de vacunación. Medicina Clínica 153, 16–27. 30621906 10.1016/j.medcli.2018.11.014

[33] San-Román-Montero JM, et al. (2019) Inpatient hospital fatality related to coding (ICD-9-CM) of the influenza diagnosis in Spain (2009–2015). BMC Infectious Diseases 19, 700. 31390988 10.1186/s12879-019-4308-5 PMC6686565

[34] Cromer D, et al. (2014) The burden of influenza in England by age and clinical risk group: A statistical analysis to inform vaccine policy. Journal of Infection 68, 363–371. 24291062 10.1016/j.jinf.2013.11.013

[35] Nielsen J, et al. (2019) European all-cause excess and influenza-attributable mortality in the 2017/18 season: Should the burden of influenza B be reconsidered? Clinical Microbiology and Infection 25, 1266–1276. 30790685 10.1016/j.cmi.2019.02.011

[36] Puig-Barberà J, et al. (2016) Influenza epidemiology and influenza vaccine effectiveness during the 2014–2015 season: Annual report from the global influenza hospital surveillance network. BMC Public Health 16(Suppl 1), 757. 27556802 10.1186/s12889-016-3378-1 PMC5001209

[37] Caini S, et al. (2018) Distribution of influenza virus types by age using case-based global surveillance data from twenty-nine countries, 1999–2014. BMC Infectious Diseases 18, 269. 29884140 10.1186/s12879-018-3181-y PMC5994061

[38] Beauté J, et al. (2015) Age-specific differences in influenza virus type and subtype distribution in the 2012/2013 season in 12 European countries. Epidemiology and Infection 143, 2950–2958. 25648399 10.1017/S0950268814003422 PMC4595855

[39] Caini S, et al. (2015) Epidemiological and virological characteristics of influenza B: Results of the global influenza B study. Influenza and Other Respiratory Viruses 9, 3–12. 26256290 10.1111/irv.12319 PMC4549097

[40] der Heiden M and Buchholz U (2017) Estimation of influenza-attributable medically attended acute respiratory illness by influenza type/subtype and age, Germany, 2001/02–2014/15. Influenza and Other Respiratory Viruses 11, 110–121. 27754611 10.1111/irv.12434 PMC5304576

[41] Puzelli S, et al. (2019) Co-circulation of the two influenza B lineages during 13 consecutive influenza surveillance seasons in Italy, 2004–2017. BMC Infectious Diseases 19, 990. 31752738 10.1186/s12879-019-4621-z PMC6873537

[42] Kalil AC and Thomas PG (2019) Influenza virus-related critical illness: Pathophysiology and epidemiology. Critical Care 23, 258. 31324202 10.1186/s13054-019-2539-x PMC6642581

[43] Wong KC, Luscombe GM and Hawke C (2019) Influenza infections in Australia 2009–2015: Is there a combined effect of age and sex on susceptibility to virus subtypes? BMC Infectious Diseases 19, 42. 30630435 10.1186/s12879-019-3681-4 PMC6327581

